# *Trichinella spiralis* serine proteinase enhances antibody-dependent cell-mediated (ADCC) killing of newborn larvae by driving M1 polarization via the NF-κB pathway

**DOI:** 10.1051/parasite/2026020

**Published:** 2026-04-17

**Authors:** Jin Yi Wu, Ru Zhang, Yao Zhang, Xin Zhuo Zhang, Dong Mei Xu, Ruo Dan Liu, Shao Rong Long, Zhong Quan Wang, Jing Cui

**Affiliations:** 1 Department of Parasitology, School of Basic Medical Sciences, Zhengzhou University Zhengzhou 450001 China; 2 Department of Clinical Nutrition, Third Affiliated Hospital of Zhengzhou University Zhengzhou 450052 China

**Keywords:** *Trichinella spiralis*, Serine proteinase, Macrophage, M1 polarization, ADCC

## Abstract

Previous studies have shown that recombinant *Trichinella spiralis* serine proteinase (rTsSPc) promoted larval invasion of the gut epithelium, but its regulatory role on macrophage polarization is not clear. Immunofluorescence assay (IFA) confirmed specific rTsSPc’s binding to RAW264.7 macrophages. The results of qPCR, Western blot, ELISA, and flow cytometry showed that rTsSPc significantly upregulated M1 macrophage markers (iNOS and CD86) and pro-inflammatory cytokines (TNF-α and IL-6), but not M2 markers (Arg1, CD206) and anti-inflammatory cytokines (IL-10, TGF-β). However, intestinal infective larvae (IIL) excretory-secretory antigens (ESAs) induced M2 polarization. Western blot revealed that rTsSPc activated the classical NF-κB pathway, as evidenced by increased phosphorylation levels of IKKβ, IκB-α, and NF-κB p65. Pretreatment of macrophages with NF-κB inhibitor pyrrolidine dithiocarbamate (PDTC) effectively suppressed rTsSPc-induced M1 polarization, decreased pro-inflammatory cytokine secretion, and reduced nitric oxide (NO) production. Functionally, rTsSPc-induced M1 polarization significantly enhanced antibody-dependent cell-mediated cytotoxicity (ADCC) of macrophages killing newborn larvae, but PDTC pretreatment resulted in a 41.62% reduction in cytotoxicity. Our results showed that rTsSPc bound specifically to macrophages and induced M1 polarization by activating the classical NF-κB pathway, thereby enhancing macrophage-mediated ADCC killing of newborn larvae. The findings indicated that TsSPc might strengthen host protective immunity via ADCC killing of larvae, and TsSPc may be considered a potential candidate antigen for developing anti-*Trichinella* vaccines.

## Abstract (fr)

Des études antérieures ont montré que la sérine protéase recombinante de *Trichinella spiralis* (rTsSPc) favorisait l’invasion larvaire de l’épithélium intestinal, mais son rôle régulateur sur la polarisation des macrophages reste incertain. Un test d’immunofluorescence (IFA) a confirmé la liaison spécifique de la rTsSPc aux macrophages RAW264.7. Les résultats des analyses qPCR, Western blot, ELISA et cytométrie de flux ont montré que la rTsSPc induisait une surexpression significative des marqueurs des macrophages M1 (iNOS et CD86) et des cytokines pro-inflammatoires (TNF-α et IL-6), mais pas des marqueurs M2 (Arg1, CD206) ni des cytokines anti-inflammatoires (IL-10, TGF-β). En revanche, les antigènes d'excrétion-sécrétion (ESA) des larves infectieuses intestinales (IIL) induisaient une polarisation M2. Le Western blot a révélé que la rTsSPc activait la voie NF-κB classique, comme en témoignent l'augmentation des niveaux de phosphorylation d'IKKβ, d'IκB-α et de NF-κB p65. Le prétraitement des macrophages avec l'inhibiteur de NF-κB, le PDTC, a efficacement supprimé la polarisation M1 induite par la rTsSPc, diminué la sécrétion de cytokines pro-inflammatoires et réduit la production d'oxyde nitrique (NO). Sur le plan fonctionnel, la polarisation M1 induite par rTsSPc a significativement renforcé la cytotoxicité cellulaire dépendante des anticorps (ADCC) des macrophages, entraînant la destruction des larves nouvelles-nées. Cependant, un prétraitement au PDTC a induit une réduction de 41,62 % de cette cytotoxicité. Ces résultats ont montré que rTsSPc se lie spécifiquement aux macrophages et induit une polarisation M1 en activant la voie NF-κB classique, renforçant ainsi l'ADCC des macrophages et leur capacité à détruire les larves nouvelles-nées. Ces observations indiquent que TsSPc pourrait renforcer l'immunité protectrice de l'hôte via l'ADCC et pourrait être considéré comme un antigène candidat potentiel pour le développement de vaccins anti-*Trichinella*.

## Introduction

Trichinellosis is a globally prevalent zoonotic parasitic disease caused by *Trichinella spiralis*. It is transmitted to humans through the consumption of raw or undercooked meat contaminated with muscle larvae (ML) [32]. This disease not only causes clinical symptoms such as myalgia, fever, and eosinophilia in trichinellosis patients, but also leads to economic losses in the pig industry due to pork food safety. Currently, the prevention and control of trichinellosis rely primarily on meat safety inspections and anthelmintic drugs, while no approved prophylactic anti-*Trichinella* vaccines are available at present, highlighting an urgent need for effective control strategies of *Trichinella* infection [50, 56].

The life cycle of *T. spiralis* involves two developmental stages: the intestinal stage and the muscle stage. Intestinal infective larvae (IIL) are critical for invading gut mucosa and establishing the infection. After being ingested by the host, ML are activated into IIL by bile and intestinal contents and then invade the intestinal epithelium to develop into adult worms [4]. This invasive process is mainly mediated by proteases present in excretory-secretory antigens (ESAs) released by IIL; these proteases play key roles in hydrolyzing the extracellular matrix and tight junctions, disrupting intestinal epithelial integrity, and modulating host immune responses [7, 9, 35, 47]. Serine proteases, as a large family of proteases present in ESAs, are involved in larval tissue penetration, molting, and immune evasion, making them potential targets for vaccine development [24, 49].

A serine proteinase (TsSPc; GenBank no.: U62659.1) was identified from IIL worm surface proteins and ESAs [20]. TsSPc is a secretory protease and is capable of binding to gut receptors for activated protein C kinase 1 (RACK1) and cytokeratin 8 (CK8), activating the MAPK/ERK1/2 and RhoA/ROCK1 signaling pathways, and reducing tight junction (TJ) proteins and disrupting gut epithelial integrity, consequently mediating larval invasion of the host intestinal epithelium [36, 37, 58]. Additionally, rTsSPc also specifically binds to the phosphoglycerate mutase family member 5 (PGAM5) receptor in the gut epithelium, triggering gut epithelial apoptosis and compromising gut barrier function, and ultimately facilitating larval invasion [24]. However, whether TsSPc modulates macrophage polarization and enhances the antibody-dependent cell-mediated cytotoxicity (ADCC) of macrophages is not reported in the literature. As core innate immune cells, macrophages display two distinct polarization phenotypes: M1 (pro-inflammatory) and M2 (anti-inflammatory/reparative) [28]. M1 macrophages secrete pro-inflammatory cytokines (TNF-α and IL-6) and nitric oxide (NO) to eliminate pathogens, whereas M2 macrophages participate in tissue repair and immune regulation [5]. In anti-parasitic immunity, macrophage polarization is critical for parasite clearance. Additionally, M1 macrophage-mediated ADCC is a key effector mechanism for killing *T. spiralis* newborn larvae (NBL) [27, 54].

Macrophages are pivotal innate immune cells that play crucial roles in recognizing and eliminating pathogens, modulating inflammatory responses, and bridging innate and adaptive immunity. These cells exhibit functional plasticity and polarize into two distinct phenotypes in response to different stimuli: classically activated M1 macrophages and alternatively activated M2 macrophages. M1 macrophages are characterized by high expression of inducible nitric oxide synthase (iNOS) and surface marker CD86, and secrete pro-inflammatory cytokines (TNF-α and IL-6), which exert direct cytotoxic effects on pathogens and enhance anti-parasitic immunity [3, 40]. In contrast, M2 macrophages express arginase-1 (Arg1) and CD206, and secrete anti-inflammatory cytokines including IL-10 and transforming growth factor-β (TGF-β), participating in tissue repair, immune regulation, and pathogen immune evasion. In anti-*T. spirali*s immunity, macrophage polarization is closely associated with parasite clearance: M1 polarization enhances the ADCC killing of NBL, a key effector mechanism for reducing parasite burden, while M2 polarization facilitates parasite colonization in host [48].

NF-κB is widely recognized as a core driver of M1 polarization, as it robustly induces pro-inflammatory gene expression upon stimulation, such as via lipopolysaccharides (LPSs). When exposed to pathogen-associated molecular patterns (PAMPs) or pro-inflammatory cytokines, macrophages rapidly activate the classical NF-κB pathway. The activated p65/p50 heterodimer translocates to the nucleus, where it directly binds and transactivates genes encoding M1 effector molecules. Nuclear translocation of NF-κB p65 is itself a hallmark of M1 macrophages [10, 14]. Therefore, the NF-κB pathway forms a central axis of M1 polarization, linking signal input (TLR agonists), core transcription factor activation (NF-κB p65/p50), and functional output (M1 effector molecules). Inhibition of NF-κB pathway activity with pharmacological inhibitors, gene knockout, or RNA interference markedly attenuates M1 polarization [19].

Several *T. spiralis* ESA proteins have been reported to regulate macrophage polarization to either facilitate parasitic infection or elicit host protective immunity [21]. A *T. spiralis* dipeptidyl peptidase 1 promoted M2 polarization to suppress macrophage cytotoxicity [48], whereas *T. spiralis* galectin drove M1 polarization to enhance ADCC [41]. A *T. spiralis* cathepsin L, a key protease at the process of gut invasion, induced macrophage M1 polarization via activating the classical NF-κB pathway. Notably, this M1 polarization significantly enhanced the macrophage cytotoxicity killing of the NBL via ADCC, which elicited host’s protective immunity [22]. Another *T. spiralis* galectin also enhanced macrophage ADCC by driving M1 polarization and increasing killing larval effects [54]. However, whether TsSPc modulates macrophage polarization and its impact on ADCC remains unclear.

The aim of this study was to investigate the effect of recombinant TsSPc (rTsSPc) on macrophage polarization. We assessed rTsSPc’s binding to macrophages, and the expression levels of M1/M2 polarization-related markers and cytokines. The NF-κB signaling pathway in rTsSPc driving macrophage polarization and its functional role for ADCC to kill NBL are also ascertained. Our results provide an experimental basis for further elucidating the immunomodulatory mechanism of TsSPc and using it as a candidate vaccine target molecule.

## Materials and methods

### Ethics

All animal experimental projects were authorized by the Life Science Ethics Committee of Zhengzhou University (No. ZZUIRB GZR 2023-1397).

### *Trichinella* species, experimental animals and cells

The *T. spiralis* isolate (ISS534) used in this research was obtained from a naturally infected domestic pig in Henan Province, China and maintained by serial passage in BALB/c mice [2]. Female mice, 4–6 weeks old, were purchased from the Experimental Animal Center of Zhengzhou University. RAW264.7 macrophages were purchased from the Cell Bank of the Chinese Academy of Sciences, and the cells were incubated at 37 °C in 5% CO_2_ with DMEM containing 10% fetal bovine serum (FBS; Gibco, Waltham, MA, USA), 100 U/mL penicillin, and 100 μg/mL streptomycin [54].

### Collection of diverse *T. spiralis* stages and preparation of larval ESAs

ML were collected by artificially digesting *T. spiralis-*infected mouse skeletal muscles at 42 days post infection (dpi) [17]. ML were activated into IIL by 5% swine bile from a slaughter house at 37 °C for 2 h [44]. After washing the IIL with sterile physiological saline and serum-free RPMI 1640 medium (100 U penicillin/mL and 100 μg/mL streptomycin), the IIL were cultured at 37 °C and 5% CO_2_ for 18 h. The culture supernatant was concentrated using an Amicon Ultra-3 centrifugal filtration device (MW cut-off value: 3 kDa) and centrifuged at 4 °C and 5,000× *g* for 3 h. The IIL ESAs were collected and stored at −80 °C until use [37]. At 6 dpi, the adults worms (AWs) were collected from the small intestine, and NBLs were obtained by culturing adult worms at 37 °C, 5% CO_2_, serum-free 1640 medium for 72 h [12].

### rTsSPc preparation

The complete TsSPc cDNA sequence was amplified by PCR and cloned into the pQE-80L carrying a His-tag at the N-terminus (Novagen, Glendale, CA, USA). The recombinant plasmid pQE-80L/TsSPc was transformed into *Escherichia coli* BL21 (Novagen). The expression of rTsSPc was induced with 0.5 mM IPTG at 37 °C for 6 h. The rTsSPc protein was purified using an Ni-NTA His-tag affinity kit (Sangon Biotech, Shanghai, China). The expression of rTsSPc was analyzed and identified by SDS-PAGE and Western blot [37].

### CCK-8 assay of RAW264.7 cell viability

The effect of rTsSPc and IIL ESA on the viability of RAW264.7 macrophages was assessed using a Cell Counting Kit-8 (CCK-8; Epizyme Biotech, Shanghai, China) [18]. Briefly, macrophage RAW264.7 cells were seeded at a density of 5 × 10^3^ cells per well in 96-well plates and cultured in high-glucose Dulbecco’s Modified Eagle Medium (DMEM; Servicebio, Wuhan, China) supplemented with 100 U/mL penicillin, 100 μg/mL streptomycin, and 10% FBS (Gibco) [25]. Different concentrations of rTsSPc and IIL ESA were added to the culture medium, and the macrophages were co-cultured at 37 °C with 5% CO_2_ for 24 and 48 h. Subsequently, 10 μL of CCK-8 reagent was added to each well and incubated at 37 °C for 2 h. The absorbance was measured at 450 nm using a multimode reader. Cell viability was calculated as a percentage, using the formula [48, 54]:

$$\text{Cell viability}\;\left(\%\right)=\left[\frac{\left(\mathrm{OD}\;\text{value of test group}-\text{blank control}\;\mathrm{OD}\;\text{value}\right)}{\left(\text{DMEM control}\;\mathrm{OD}\;\text{value}-\text{blank control}\;\mathrm{OD}\;\text{value}\right)}\right]\times 100\%$$

### Immunofluorescence assay (IFA) of rTsSPc binding with RAW264.7 cells

IFA was performed to assess the binding of rTsSPc with RAW264.7 macrophage as described previously [48]. In brief, macrophages were cultured on glass coverslips in 24-well plates until they reached 80% confluence. The cells were washed three times with PBS, fixed with 4% glutaraldehyde for 20 min at room temperature, and then incubated with 25 μg/mL rTsSPc or IIL ESA at 37 °C for 2 h [21]. The cells were then blocked with 5% goat serum at 37 °C for 1 h. Subsequently, the cells were incubated with different sera (1:20 dilutions; anti-rTsSPc serum, *T. spiralis*-infected mouse serum or pre-immune serum) at 37 °C for 1 h. CY3-conjugated anti-mouse IgG (1:100; Servicebio) was used as the secondary antibody. 4’,6-Diamidino-2-phenylindole dihydrochloride (DAPI) was used to stain the cell nuclei blue. Fluorescence signals were observed under a fluorescence microscope (Olympus, Tokyo, Japan) [47].

### qPCR, Western blot, and ELISA evaluation of rTsSPc-induced RAW264.7 macrophage M1 polarization

The cell suspension of RAW264.7 macrophages was seeded into 6-well plates and incubated for 1 h. Then, the cells were stimulated with 25 μg/mL rTsSPc for 24 h. After stimulation, the cells were collected, and RNA was extracted [33]. The RNA was reverse-transcribed into cDNA, and qPCR was performed using the cDNA as a template to assess transcriptional levels of M1 (iNOS, IL-6, and TNF-α) and M2-related factors (Arg1, IL-10, and TGF-β). LPS (200 ng/mL) was used as the M1 positive control, IL-4 (20 ng/mL) as the M2 positive control, and DMEM as the negative blank control [48]. IIL ESAs were used as the *T. spiralis* control. β-actin was used as the endogenous control, and the 2^-ΔΔCt^ method was used to analyze the relative gene expression levels [3].

Additionally, Western blot analysis was performed by using soluble proteins from RAW264.7 macrophages treated with rTsSPc for 24 h [41, 42]. Primary antibodies included rabbit anti-mouse iNOS, mouse anti-mouse Arg1, and anti-mouse β-actin. Secondary antibodies were HRP-conjugated anti-rabbit and anti-mouse antibodies.

The culture supernatant of RAW264.7 macrophages was centrifuged at 1,000× *g*, at 4 °C for 10 min, and was collected. Macrophage-secreted inflammatory cytokines (TNF-α, IL-6, TGF-β, and IL-10) were measured by a double-antibody sandwich ELISA kit (eBioscience, San Diego, CA, USA) [46, 52]. Cytokine concentrations were expressed in nanograms per milliliter (ng/mL).

### Flow cytometry

After RAW264.7 macrophages were pretreated with inhibitor PDTC, the macrophages were washed three times with PBS and resuspended in FACS buffer (PBS, 0.1 % BSA, and 0.5 mM EDTA) for staining [48]. The cells were first blocked with anti-mouse CD16/CD32 antibody (mouse Fc blocker, 1:100, eBioscience) on ice for 20 min for flow cytometry. Subsequently, the cells were incubated on ice in the dark with fluorochrome-conjugated antibodies for 20 min, followed by washing with FACS buffer and centrifugation at 1,600× *g* for 5 min. Macrophages were identified using FITC-conjugated anti-F4/80 antibody (eBioscience) and PerCP-Cyanine5.5-conjugated anti-CD11b antibody (eBioscience). PE-conjugated anti-CD86 antibody was served as an M1 marker, and APC-conjugated anti-CD206 antibody (eBioscience) served as an M2 marker. For intracellular molecule analysis, the macrophages were permeabilized with the intracellular fixation/permeabilization buffer kit (Elabscience, Wuhan, China) and stained intracellularly with APC-conjugated anti-CD206 [54]. Finally, the cell samples were analyzed on a BD FACSCanto flow cytometer (BD Biosciences, San Jose, CA, USA), and the data were further processed using FlowJo software (Becton Dickinson and Company, Portland, OR, USA) [31].

### Western blot of the classical NF-κB signaling pathway in rTsSPc-driven macrophage M1 polarization

The effect of rTsSPc on the NF-κB signaling pathway in rTsSPc-treated RAW264.7 macrophages was investigated [22]. After stimulation for 3 h, changes in p-IKKβ, IKKβ, p-IκB-α, and IκB-α were assessed. After stimulation for 24 h, changes in p-NF-κB p65 and NF-κB p65 were also evaluated by Western blot. Briefly, cellular soluble proteins were prepared and separated by 10% polyacrylamide gel, transferred to polyvinylidene difluoride (PVDF) membranes, blocked with 5% skimmed milk in Tris-buffered saline with Tween 20 (TBST) for 2 h. The membrane was cut into strips, and the strips were incubated with primary antibodies in TBST overnight at 4 °C as follows: antibodies against p-IKKβ, IKKβ, p-IκB-α, IκB-α, p-NF-κB p65, and NF-κB p65 (1:1000, Abmart, Shanghai, China), with corresponding secondary antibodies being goat anti-rabbit IgG (1:5000). β-actin was used as the internal control [29, 34, 41]. Omni-ECLTm reagents (Epizyme, Shanghai, China) were used to visualize the reactive bands, and the relative intensity of each band was evaluated using Image J software (National Institutes of Health, Bethesda, MD, USA) [43].

### qPCR, Western blot, and ELISA detection of inhibitor suppressing rTsSPc-driven macrophage M1 polarization and NF-κB pathway activation

To evaluate the effect of NF-κB inhibitor PDTC suppressive on the NF-κB pathway activation in rTsSPc-treated RAW264.7 macrophages, expression levels of iNOS, IL-6, and TNF-α, and NF-κB p65 phosphorylation levels were assessed by qPCR or Western blot [25, 39]. Briefly, RAW264.7 macrophage cells were pre-incubated with 150 μM PDTC for 2 h, and then stimulated with 25 μg/mL of rTsSPc for an additional 24 h. LPS at 200 ng/mL was used as a positive control. After incubation, total RNA was extracted from the RAW264.7 cells for qPCR analysis (Table 1), and cellular proteins were extracted for Western blot analysis [8, 35]. Cell culture supernatants were obtained to assay cytokine levels (TNF-α and IL-6) by ELISA [57].

**Table 1:** Specific primer sequences of macrophage markers and cytokines for qPCR analysis.

Genes	Sequences (5′–3′)	GenBank No.
TNF-α	F: CCCTCACACTCAGATCATCTTCT	NM_013693.3
(Mouse)	R: GCTACGACGTGGGCTACAG	
TGF-β	F: AGCAACAATTCCTGGCGTTACCT	NM_011577.2
(Mouse)	R: CCTGTATTCCGTCTCCTTGGTTCA	
IL-6	F: TACCACTTCACAAGTCGGAGGC	NM_001314054.1
(Mouse)	R: CTGCAAGTGCATCATCGTTGTTC	
IL-10	F: CCCTTTGCTATGGTGTCCTT	NM_010548.2
(Mouse)	R: TGGTTTCTCTTCCCAAGACC	
iNOS	F: GAGAGACAGGGAAGTCTGAAGCAC	NM_010927.4
(Mouse)	R: CCAGCAGTAGTTGCTCCTCTTC	
Arg1	F: CATTGGCTTGCGAGACGTAGAC	NM_007482.3
(Mouse)	R: GCTGAAGGTCTCTTCCATCACC	
β-actin	F: CTACCTCATGAAGATCCTGACC	NM_007393.5
(Mouse)	R: CACAGCTTCTCTTTGATGTCAC	

### Assay of NO production from rTsSPc-stimulated macrophages

RAW264.7 macrophages were incubated with 25 μg/mL of rTsSPc for 24 h. LPS (200 ng/mL) was used as the M1 positive control, IL-4 (20 ng/mL) as the M2 positive control, and IIL ESAs as the rTsSPc positive control. Cell culture supernatants were collected and mixed with 50 μL of Griess reagent I and II [30]. The mixture was incubated, and the absorbance was measured at 540 nm. A standard curve was generated using NaNO_2_ solutions of varying concentrations (0, 1, 2, 5, 10, 20, 40, 60, and 100 μM) to determine the concentration of NO in the supernatants [48].

### ADCC assay

The macrophage killing of NBL through the ADCC test was performed as described previously [53, 57]. The RAW264.7 macrophages (1 × 10^4^ cells/well) were cultured in a 96-well plate and pre-incubated with 25 μg/mL of rTsSPc at 37 °C for 24 h. After renewal of the medium, 100 NBLs were added to the medium supplemented with 1:100 dilutions of *T. spiralis-*infected mouse serum. After incubation at 37 °C for 36 h, larval viability was assessed on their morphology and activity. Cytotoxicity was calculated as the percentage of dead NBL or NBL adhered by macrophages to the total larvae observed in each assay [1, 11].

Moreover, to evaluate the suppressive effect of p-NF-κB p65-specific inhibitor PDTC on macrophage cytotoxicity, RAW264.7 macrophages were first pretreated with 150 μM PDTC for 2 h, incubated with 25 μg/mL of rTsSPc for 24 h, and then used for the ADCC test [25].

### Statistical analysis

All statistical analyses were performed using GraphPad Prism version 9.5.0 (GraphPad Software, San Diego, CA, USA). The data are presented as mean ± standard deviation (SD). One-way ANOVA, Student’s *t*-test and χ^2^ tests were applied to assess the statistical differences. *P*-values < 0.05 were regarded as statistically significant.

## Results

### rTsSPc had no obvious cytotoxicity on RAW264.7 cells

**Figure 1:**
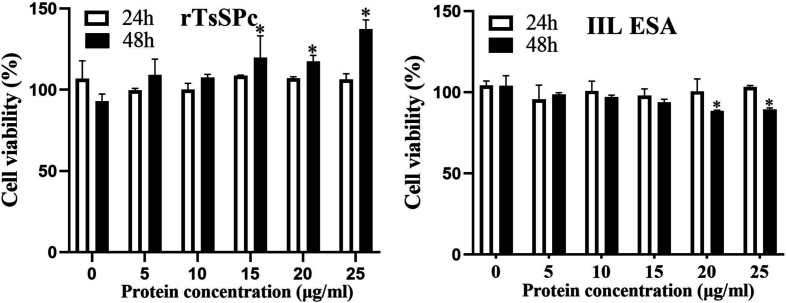
CCK-8 assay of rTsSPc and IIL ESA effects on RAW264.7 cell viability. rTsSPc or IIL ESA (5, 10, 15, 20, and 25 μg/mL) were co-cultured with RAW264.7 macrophages for 24 and 48 h, and the effect of rTsSPc on cell viability was assessed. The OD_450_ values were measured by the SpectraMax i3X (Molecular Devices, San Jose, CA, USA). The data from three independent tests are exhibited as the mean ± SD. *p < 0.05 in comparison with the PBS group.

The effect of rTsSPc and IIL ESAs on RAW264.7 cellular viability was evaluated after incubation for 24 and 48 h. Various concentrations of rTsSPc proteins did not have obvious effects on RAW264.7 cell viability after incubation for 24 h. However, 15–25 μg/mL of rTsSPc increased cell viability after incubation for 48 h (*F*_24h_ = 5.420, *p* = 0.0078; *F*_48h_ = 12.60, *p* = 0.0002). Additionally, 20 and 25 μg/mL IIL ESA incubation for 24 h had no evident effect on cell viability (*F*_24h_ = 2.382, *p* = 0.1012) (Fig. 1). Therefore, 25 μg/mL of rTsSPc and IIL ESA were used to incubate with RAW264.7 cells for 24 h in subsequent experiments.

### Binding of rTsSPc with RAW264.7 macrophages

**Figure 2:**
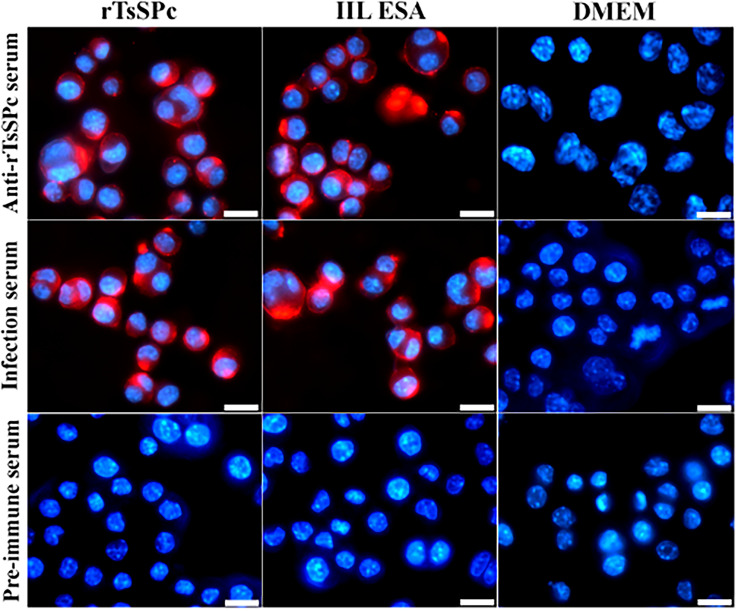
IFA detection of rTsSPc binding with RAW264.7 macrophages. The RAW264.7 macrophages were incubated with rTsSPc protein at 37 °C for 2 h. Different sera (1:20; anti-rTsSPc serum, infected serum, or pre-immune serum) were used as the primary antibody, and CY3-labeled anti-mouse IgG (1:100) was used as the secondary antibody. Cell nuclei were stained blue with DAPI. The specific binding of rTsSPc to the RAW264.7 macrophages was observed under a fluorescence microscope. Each test had three replicates. Scale bar: 5 μm.

IFA was performed to investigate the binding of rTsSPc with macrophages. The results showed that after incubation with rTsSPc, red fluorescence was detected on the macrophage using anti-rTsSPc serum and infection serum (Fig. 2). The results indicate that rTsSPc specifically binds to macrophages, confirming direct interaction between rTsSPc and macrophages, laying the molecular foundation for its immunomodulatory effects on macrophages.

### rTsSPc induced RAW264.7 macrophage M1 polarization

**Figure 3:**
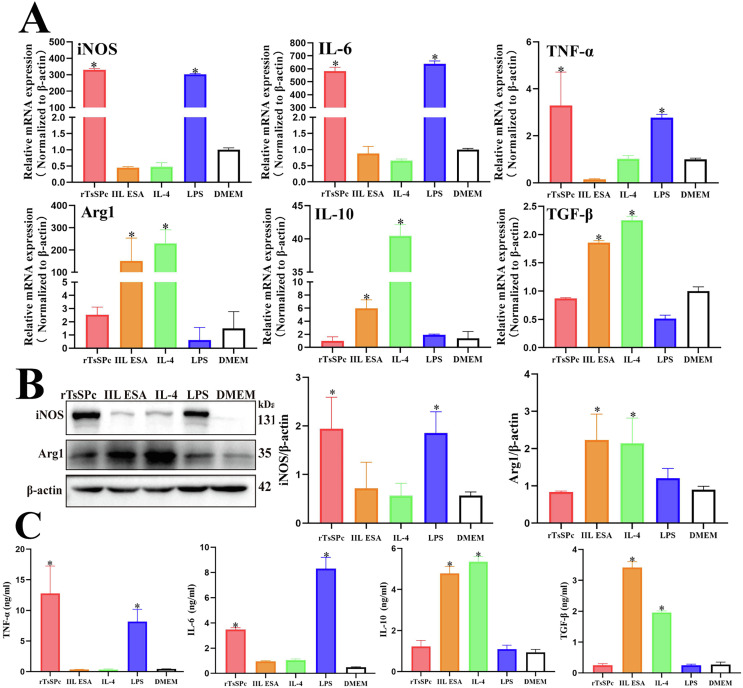
Expression levels of M1/M2-related factors in rTsSPc-treated RAW264.7 macrophages. A: qPCR assay of mRNA expression levels of M1/M2-related factors. RAW264.7 macrophages were treated with rTsSPc for 24 h. rTsSPc drove macrophage M1 polarization and increased mRNA levels of M1-related factors. M1-related genes include iNOS, IL-6, and TNF-α, while M2-related genes include Arg1, IL-10, and TGF-β. Their mRNA expression levels were calculated using the Ct^(2−ΔΔCt)^ method, with β-actin serving as the housekeeping gene. B: Western blot of expression levels of iNOS and Arg1 in RAW264.7 cells. C: ELISA detection of cytokine (TNF-α, IL-6, TGF-β, and IL-10) produced from rTsSPc-stimulated RAW264.7 macrophages. Each test had three replicates. * *p* < 0.05 compared to the DMEM group.

After RAW264.7 cells were treated with rTsSPc, qPCR results of transcriptional levels of M1/M2-related factors revealed that rTsSPc upregulated the expression of M1-related genes iNOS, TNF-α, and IL-6 (*F*_iNOS_ = 5868, *p* < 0.0001; *F*_IL-6_ = 1196, *p* < 0.0001; *F*_TNF-α_ = 12.87, *p* = 0.0006). In contrast, IIL ESAs upregulated the expression of M2-related genes Arg1, IL-10, and TGF-β (*F*_Arg1_ = 12.05, *p* = 0.0008; *F*_IL-10_ = 714.2, *p* < 0.0001; *F*_TGF-β_ = 513.0, *p* < 0.0001) (Fig. 3A). These results indicate that rTsSPc promotes M1 polarization in RAW264.7 cells, while IIL ESAs promotes M2 polarization.

Western blot analysis results showed that, compared to the DMEM group, treatment of RAW264.7 cells with 25 μg/mL rTsSPc for 24 h significantly increased the expression level of iNOS (*F* = 10.38, *p* < 0.01); but treatment with IIL ESAs for 24 h significantly increased the expression level of Arg1 (*F* = 11.36, *p* < 0.01) (Fig. 3B). Expression levels of cytokines produced by rTsSPc-treated RAW264.7 were ascertained by ELISA. The ELISA results revealed that rTsSPc significantly up-regulated the expression of M1 pro-inflammatory cytokines IL-6 and TNF-α (*F*_IL-6_ = 194.8, *p* < 0.0001; *F*_TNF-α_ = 20.66, *p* < 0.0001), but rTsSPc had no obvious effect on expression levels of M2 anti-inflammatory cytokines IL-10 and TGF-β (*F*_IL-10_ = 1.300, *p =* 0.3396; *F*_TGF-β_ = 0.2079, *p* = 0.8179) (Fig. 3C). These findings suggest that rTsSPc significantly promoted pro-inflammatory macrophage M1 polarization, while IIL ESAs induced anti-inflammatory M2 polarization.

### rTsSPc increased the expression of M1 marker CD86 in macrophages

**Figure 4:**
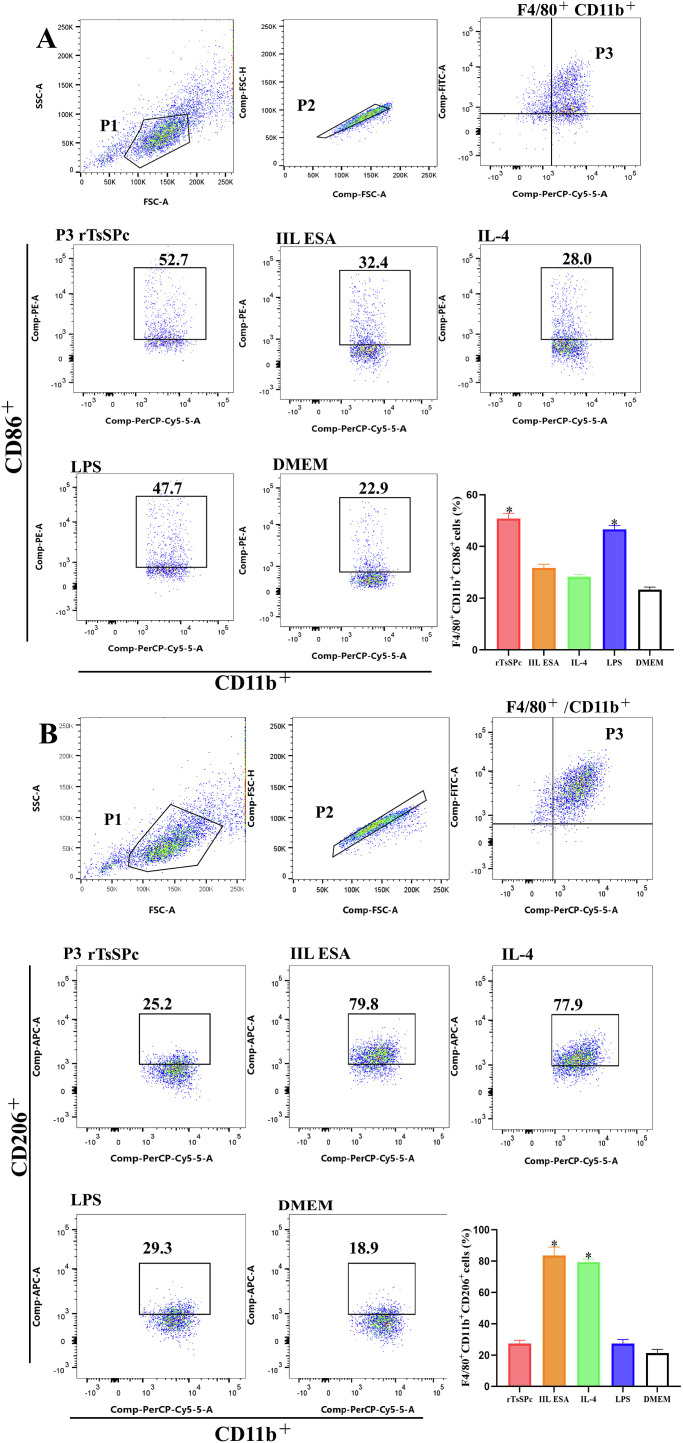
Flow cytometry of rTsSPc-induced RAW264.7 macrophage M1 polarization. Flow cytometry was performed to evaluate the rTsSPc’s effect on the polarization of RAW264.7 macrophages. **A:** The rTsSPc effect on CD86 (M1) expression in RAW264.7 macrophages was assessed, lipopolysaccharide (LPS) was used as the M1 positive control. The macrophages were identified by co-staining for F4/80 and CD11b. **B:** The impact of rTsSPc on CD206 (M2) expression was also examined, with IL-4 serving as the M2 positive control, and the same F4/80/CD11b markers were applied to identify RAW264.7 macrophages. Each test had three replicates. * *p* < 0.05 compared with the DMEM group.

RAW264.7 macrophages were labeled with F4/80 and CD11b, with CD86^+^ cells representing the M1 phenotype and CD206^+^ cells representing the M2 phenotype. Flow cytometry analysis revealed that after treatment with rTsSPc and IIL ESAs, rTsSPc increased the number of CD86^+^ cells by 117.7% (*F* = 213, *p* < 0.0001); whereas IIL ESAs increased the number of CD206^+^ cells by 290.7% (*F* = 314.2, *p* < 0.0001) (Fig. 4), compared with the DMEM group. Combined with the results of Western blot, qPCR, ELISA, and flow cytometry data, these findings indicated that rTsSPc induced RAW264.7 macrophages to polarize toward the M1 phenotype, while IIL ESAs drove macrophages to polarize toward the M2 phenotype.

### rTsSPc activated the classical NF-κB signaling pathway in M1 polarized RAW264.7 macrophages

**Figure 5:**
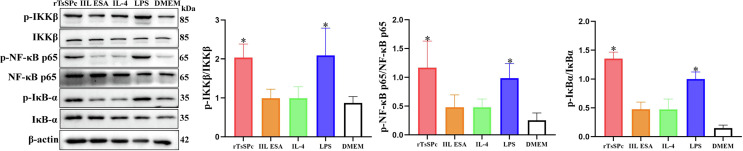
Western blot of NF-κB pathway proteins in M1 polarized RAW264.7 macrophages. RAW264.7 macrophages were treated with rTsSPc, and the expression of NF-κB signaling pathway-related proteins was assessed using Western blot analysis. The relative intensity of protein signals for p-IKKβ, p-IκB-α, and p-NF-κB p65 was quantified, and β-actin was used as the internal control. Each test had three replicates. *Compared to the DMEM group, *p* < 0.05.

Western blot results showed that, compared to the DMEM group, treatment of RAW264.7 cells with 25 μg/mL of rTsSPc did not significantly alter expression levels of IKKβ, IκB-α, and NF-κB p65 (*p >* 0.05). However, the phosphorylated forms of IKKβ (p-IKKβ), IκB-α (p-IκB-α), and NF-κB p65 (p-NF-κB p65) were significantly increased, as demonstrated by obviously elevated phosphorylation levels of IKKβ, IκB-α, and NF-κB p65 (*F*_p-IKKβ_ = 7.287, *p <* 0.01; *F*_p-NF-κB p65_ = 6.121, *p* < 0.01; *F*_p-IκB-α_ = 44.62, *p* < 0.0001) (Fig. 5). The results suggest that rTsSPc activates the classical NF-κB pathway, a core signaling cascade driving macrophage M1 polarization.

### Inhibitor PDTC suppressed rTsSPc-driven macrophage M1 polarization and NF-κB pathway activation

**Figure 6:**
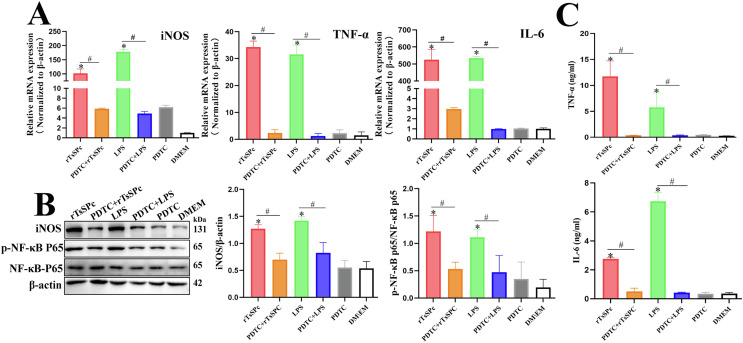
qPCR, Western blot, and ELISA detection of inhibitor (PDTC) suppressing rTsSPc-driven macrophage M1 polarization and NF-κB pathway activation. A: qPCR assay of transcription levels of iNOS, IL-6, and TNF-α. **B:** Western blot of expression levels of iNOS, NF-κB p65, and p-NF-κB p65. **C:** Contents of IL-6 and TNF-α secreted by macrophages assayed by ELISA. Each test had three replicates. * Compared with the DMEM group, *p* < 0.05; ^#^Comparison between the two groups, *p* < 0.05.

The qPCR results showed that, compared to the rTsSPc group, the transcription levels of iNOS, TNF-α, and IL-6 in the PDTC+rTsSPc group were significantly decreased after the macrophages were pretreated by PDTC (*t*_iNOS_ = 10.65, *p* = 0.0004; *t*_TNF-α_ = 21.58, *p* < 0.0001; *t*_IL-6_ = 14.91, *p =* 0.0001) (Fig. 6A), suggesting that the rTsSPc-increased transcription levels of iNOS and pro-inflammatory cytokines TNF-α and IL-6 in RAW264.7 cells are mediated through the activation of the classical NF-κB pathway. The results of Western blot analysis also showed that after PDTC pretreatment, iNOS expression levels in RAW264.7 cells were distinctly reduced, and the phosphorylation level of NF-κB p65 was obviously decreased (*t*_iNOS =_ 6.851, *p* < 0.01; *t*_p-NF-κB p65_ = 3.712, *p* < 0.05) compared to the rTsSPc group (Fig. 6B). The ELISA results revealed that expression levels of IL-6 and TNF-α in the PDTC+rTsSPc group were significantly decreased relative to the rTsSPc alone group (*t*_TNF-α_ = 6.540, *p* = 0.0028; *t*_IL-6_ = 16.42, *p* < 0.0001) (Fig. 6C). The results further indicate that rTsSPc drove macrophage M1 polarization, increased expression levels of iNOS, and cytokines IL-6 and TNF-α via the NF-κB signaling pathway. The PDTC inhibition experiment validated that rTsSPc-driven M1 polarization is NF-κB pathway-dependent, the core hypothesis of this study.

### rTsSPc increased NO production

**Figure 7:**
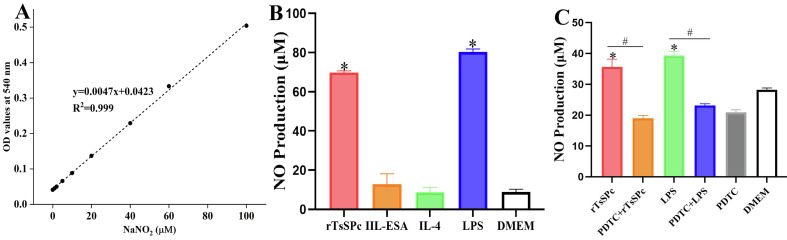
Assay of NO production in cultivated macrophage supernatant. **A:** Standard curve for NO concentration. **B:** NO production in various groups of macrophages after treatment for 24 h. **C**: NO production in macrophages pretreated with PDTC for 2 h, followed by co-culture with rTsSPc for 24 h. LPS (200 ng/mL) was used as a positive control, and IL-4 (20 ng/mL) as a negative control. Each test had three replicates. *p < 0.05 compared to the DMEM group; ^#^*p* < 0.05 between groups.

The Griess reaction was used to assess rTsSPc stimulation on RAW264.7 macrophage NO production. A standard curve of NO concentrations was drawn based on the OD values at 540 nm of a serial concentration of NaNO_2_ (Fig. 7A). The results revealed that the NO production in rTsSPc-stimulated macrophages was 6.86-fold higher than in the DMEM group (*t* = 65.50, *p* < 0.0001) (Fig. 7B). However, NO production in IIL ESA-stimulated macrophages did not show significant increases compared to the DMEM group (*t* = 1.267, *p* = 0.2739). Additionally, after macrophages were pretreated with a specific NF-κB inhibitor (PDTC) and then stimulated with rTsSPc, NO production in the PDTC-pretreated macrophages decreased by 46.60%, compared to the individual rTsSPc group (*t* = 10.73, *p* = 0.0004) (Fig. 7C). These results further indicate that rTsSPc drove macrophage M1 polarization and increased NO production of M1 macrophages by activating the classical NF-κB pathway.

### rTsSPc enhanced macrophage ADCC killing of NBL by driving macrophage M1 polarization and NF-κB pathway activation

**Figure 8:**
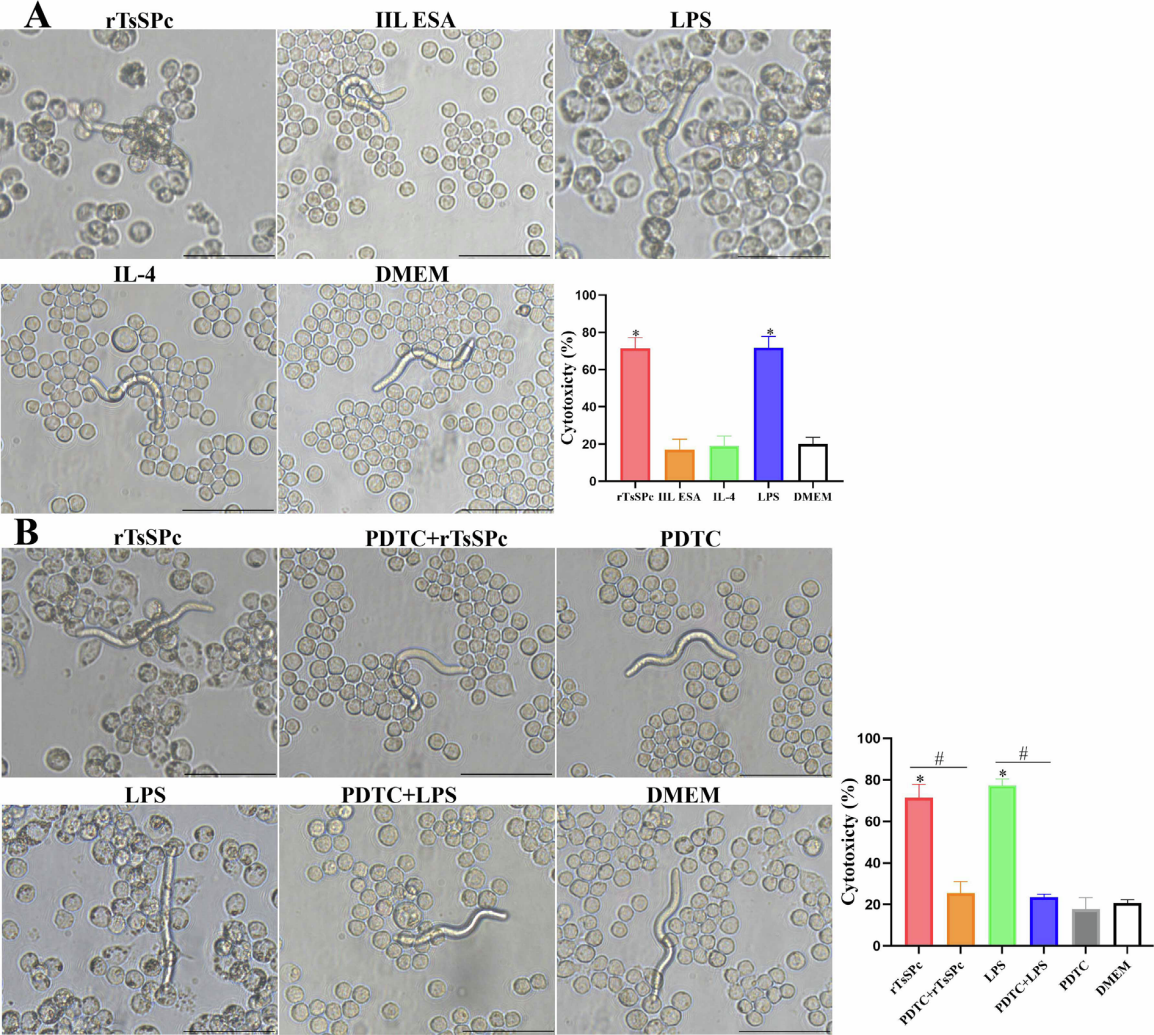
ADCC killing of *Trichinella spiralis* newborn larvae. **A:** rTsSPc enhanced macrophage cytotoxicity. RAW264.7 macrophages were first stimulated with 25 μg/mL of rTsSPc at 37 °C for 24 h, and incubated with *T. spiralis* infection serum and 100 newborn larvae (NBL) for 36 h. **B:** The macrophages were pretreated with specific p-NF-κB p65 inhibitor PDTC for 2 h, and then stimulated with rTsSPc for 24 h, finally incubated with infection serum and NBL. Each test had three replicates. ^*^p < 0.05 compared to the DMEM group. ^#^*p* < 0.05 between the two groups. Scale bar: 50 μm.

The results of the ADCC assay showed that compared to the DMEM group, more macrophages adhered to NBLs, and the macrophages’ cytotoxicity in the rTsSPc group was obviously increased (χ^2^ *=* 150.7, *p <* 0.0001) (Fig. 8A). In contrast, NBLs exhibited higher activity in the IIL ESA group with no evident cytotoxicity.

When macrophages were pretreated with the NF-κB p65-specific inhibitor PDTC and used for the ADCC assay, the NBL activity significantly increased, and the number of macrophages adhering to NBLs was obviously decreased, compared to the rTsSPc-only group, as demonstrated by a 41.62% reduction in macrophage cytotoxicity (χ^2^ = 160.5, *p* < 0.001) (Fig. 8B), indicating that PDTC inhibited rTsSPc-induced M1 polarization and NF-κB pathway activation. These results suggest that rTsSPc enhanced macrophage cytotoxicity killing of *T. spiralis* larvae via M1 polarization and NF-κB pathway activation. ADCC results had translated the mechanistic finding (M1 polarization) into functional anti-parasitic activity, confirming the role of rTsSPc in enhancing host protective immunity via ADCC.

## Discussion

The host immune system is pivotal to combating *T. spiralis* infection. During the early phase of infection, the intestinal mucosal immune system is activated to clear intestinal adult worms. Thereafter, systemic immune response is recruited to combat migrating newborn larvae. In this process, innate immune cells, especially macrophages, serve as the first line of defense against the parasite [13, 45].

Macrophages are highly plastic immune cells that differentiate into functionally distinct subtypes in response to microenvironmental signals – a process referred to as “macrophage polarization.” The classical polarization phenotypes encompass M1 (classically activated) and M2 (alternatively activated) macrophages. M1 macrophages are commonly induced by PAMPs, LPS or Th1 cytokines (IFN-γ) [38, 48]. They exhibit potent pro-inflammatory activity, marked by high expression of iNOS, TNF-α, IL-6, and IL-12. Through the release of reactive oxygen species (ROS) and reactive nitrogen species (RNS), M1 macrophages directly destroy and eradicate invading pathogens, including bacteria, viruses, and specific developmental stages of parasites [54]. M2 macrophages are primarily activated by Th2 cytokines (e.g., IL-4 and IL-13) or anti-inflammatory factors (such as IL-10 and TGF-β). They exert key roles in suppressing inflammation, facilitating tissue repair and remodeling, and regulating immune responses. Their characteristic markers include Arg1, mannose receptor (CD206), FIZZ1, and Ym1. During the muscle phase of *T. spiralis* infection, M2 macrophage activation facilitates larval encapsulation and tissue repair, yet establishes an immunosuppressive microenvironment to support the long-term survival of the parasite [21]. Thus, the dynamic balance between macrophage M1 and M2 polarization is pivotal in determining the outcome of *T. spiralis* infection. Robust early M1 polarization facilitates parasite clearance, but excessive or sustained M1 activation might result in significant tissue damage.

In this study, we aimed to investigate whether rTsSPc affects the polarization of RAW264.7 macrophages. We used endotoxin-free rTsSPc to directly stimulate macrophages, with LPS as the M1 inducer and IL-4 as the M2 inducer. IIL ESAs were used as the positive control. CCK-8 assay results demonstrated that stimulating macrophages with 5–25 μg/mL rTsSPc had no significant impact on cell viability. IFA revealed that rTsSPc binds specifically to macrophages, suggesting an interaction between rTsSPc and host macrophages. However, rTsSPc’s specific receptor on the macrophage surface was not identified. Therefore, in future studies, the properties of the TsSPc-binding receptor on macrophages need to be further characterized using, for example, co-immunoprecipitation (Co-IP), pull-down assays, and mass spectrometry [6, 36].

To further investigate whether rTsSPc induces macrophage polarization, the expression of M1/M2 macrophage effector molecules was assessed in this study. Flow cytometry results revealed that the proportion of CD86-positive cells in the rTsSPc group was significantly higher than that of the control group. CD86, a co-stimulatory molecule belonging to the B7 family, is a typical hallmark of M1 macrophages. As antigen-presenting cells (APCs), M1 macrophages can more effectively activate naïve T cells by upregulating CD86 expression, thereby initiating adaptive immune responses [23]. qPCR and Western blot results revealed that rTsSPc significantly up-regulated mRNA and protein expression of iNOS. Likewise, qPCR and ELISA results showed that rTsSPc stimulation significantly elevated both the transcription and secretion level of pro-inflammatory cytokines IL-6 and TNF-α. Nevertheless, under the same conditions of culture with rTsSPc, the expression of M2 markers showed no significant alterations. These findings indicated that rTsSPc induced macrophage M1 polarization.

The classical NF-κB signaling pathway is typically regulated by an inactive cytoplasmic complex consisting of the transcription factor NF-κB (primarily the p65/p50 heterodimer) and its inhibitory proteins from the IκB family (mainly IκB-α) [41]. Upon external cellular stimulation, the upstream IκB kinase (IKK) complex predominantly composed of IKKα and IKKβ is activated, leading to IκB-α phosphorylation. Subsequent to this activation, phosphorylated IκB-α undergoes ubiquitination and proteasomal degradation, thereby releasing the NF-κB dimer. The liberated NF-κB then rapidly translocates into the nucleus, binds to κB sites within the promoter regions of target genes, and initiates the transcription of a suite of pro-inflammatory genes. Our results showed that at 3 h after rTsSPc stimulation, the IKKβ phosphorylation level (p-IKKβ) increased significantly. Following IKKβ activation, the IκB-α phosphorylation level (p-IκB-α) was also rapidly elevated. Concurrently with IκB degradation, the phosphorylation level of the NF-κB p65 subunit (p-NF-κB p65) increased markedly [18]. To further validate these pathway changes in rTsSPc-treated macrophages, we used the NF-κB pathway inhibitor PDTC, which effectively blocks NF-κB nuclear translocation and DNA-binding activity. Our results confirmed that after pretreatment of macrophages with PDTC, rTsSPc did not effectively induce the upregulation of both the mRNA and protein levels of M1 markers (iNOS, TNF-α, and IL-6). Additionally, PDTC significantly attenuated rTsSPc-induced NO production capacity. Collectively, these results indicate that rTsSPc induced macrophage M1 polarization in a manner dependent on the activation of the classical NF-κB signaling pathway.

ADCC serves as a key immune effector mechanism during parasitic infections [26]. When specific antibodies (typically IgG or IgE) against parasite surface antigens bind to the parasite, their Fc regions are recognized by immune cells (e.g., macrophages, eosinophils and neutrophils) that express the corresponding Fc receptors (FcR). This interaction triggers effector cells to release cytotoxic mediators [1, 55]. A critical phase of *T. spiralis* infection is NBL migration through blood from the small intestine to skeletal muscles. Effective clearance of NBL from the blood circulatory system is critical to reduce the parasite burden and mitigate tissue damage. By using *in vitro* co-culture experiments, we assessed the ability of rTsSPc-activated macrophages to kill NBL. Compared to normal macrophages, the rTsSPc pre-stimulated macrophages significantly enhanced their ability to kill NBL. The pro-inflammatory cytokines (TNF-α and IL-6) secreted by M1 macrophages foster an inflammatory microenvironment that facilitated AW clearance from the gut, reduced NBL production, and thereby decreased the larval burdens in skeletal muscles [54]. These findings suggested that TsSPc could be considered a potential candidate target antigen for developing anti-*Trichinella* vaccines.

The balance of macrophage M1/M2 polarization determines the outcome, inflammatory severity, and tissue homeostasis of *T. spiralis* infection. Studies have confirmed that macrophages in key immune tissues showed an M1 phenotype in early *T. spiralis* infection, which boosted inflammation and correlated with host anti-parasite resistance [45]. During the intestinal phase of *T. spiralis* infection, macrophages are recruited and activated at infection sites to respond directly to invading larvae and adults [16] (Lee *et al.*, 1983). rTsSPc induced macrophage M1 polarization and markedly enhanced the ADCC effect, providing the direct experimental evidence that M1 polarization mediated macrophage’s anti-*T. spiralis* activity to kill migrating NBL. Additionally, M1-secreted pro-inflammatory cytokines (TNF-α and IL-6) created a parasite-hostile microenvironment, recruited the immune cells to directly damage the nematode [51]. Simultaneously, *T. spiralis* has evolved systematic immune evasion strategies. The parasite forms an anatomical seclusion state in host cells and releases immunomodulatory molecules via ESA to actively modulate host immune response, with its robust immunosuppressive capacity being vital for long-term coexistence with the host [15]. Specifically, rTsSPc triggers the host’s M1 polarization-centered protective immune attack, while the parasite counteracts this defense through evolutionarily developed immune evasion strategies.

Although this study yielded some novel insights into TsSPc functions, it still has several limitations. The rTsSPc’s role on macrophage M1/M2 polarization was verified only in murine RAW264.7 macrophage cell line *in vitro*. The *in vivo* validation of rTsSPc driving M1 polarization is needed in *T. spiralis*-infected animals. The *in vivo* complex immune microenvironment characterized by crosstalk among multiple immune cells and tissue factors cannot be fully simulated in the *in vitro* monoculture system, so the regulatory role of TsSPc on macrophage polarization and ADCC-mediated larval killing require further *in vivo* verification. Moreover, our results indicate the specific binding of rTsSPc to macrophages, but the cognate macrophage surface receptor for TsSPc remains unidentified. These issues should be clarified in future research.

In conclusion, rTsSPc activated the classical NF-κB pathway of macrophages, up-regulated M1 phenotypic markers (iNOS, CD86), and increased mRNA and protein levels of pro-inflammatory cytokines (TNF-α, IL-6), confirming that rTsSPc drives macrophage polarization toward the M1 phenotype. Consistently, rTsSPc-activated M1 macrophages exhibited markedly increased NO synthesis and secretion. The rTsSPc-induced M1 polarization significantly enhanced macrophage cytotoxicity, and notably ‌strengthened ‌their ability to destroy NBL through ADCC. rTsSPc-triggered M1 polarization at the early stage of *T. spiralis* infection serves as an active, effective defensive strategy for the host immune system to limit the establishment of the nematode infection. These findings show that TsSPc enhanced host protective immunity against *T. spiralis* by enhancing the ADCC killing of NBL, highlighting its potential as a candidate antigen for developing prophylactic anti-*Trichinella* vaccines.
